# Leishmaniasis in Chaparé, Bolivia

**DOI:** 10.3201/eid1504.081257

**Published:** 2009-04

**Authors:** Ernesto Rojas, Rudy Parrado, Raúl Delgado, Richard Reithinger, Ana L. Garcia

**Affiliations:** Universidad Mayor de San Simón, Cochabamba, Bolivia (E. Rojas, R. Parrado, R. Delgado, A.L. Garcia); London School of Hygiene and Tropical Medicine, London, UK (R. Reithinger); George Washington University School of Medicine and Health Science, Washington, DC, USA (R. Reithinger)

**Keywords:** Leishmaniasis, Leishmania (Viannia) braziliensis, control, Bolivia, letter

**To the Editor:** In Bolivia, most cases of leishmaniasis are caused by *Leishmania* (*Viannia*) *braziliensis* (*1*). The parasite is transmitted zoonotically by several sandfly species and, when transmitted to humans, may cause cutaneous leishmaniasis (CL), and potentially, mucosal leishmaniasis (ML) (*2*).

Data on the prevalence and effects of CL in Bolivia have been scarce, even though anecdotal and official reports indicate a dramatic increase in the number of human CL cases in Bolivia in the past decade (*1*,*3*). Also, although CL was originally a sylvatic disease in Bolivia, some evidence indicates that the transmission cycle has adapted to the peridomestic habitat. However, this evidence is largely based on individual case reports. No information is available on parasite species, vectors, and reservoirs in such a peridomestic transmission cycle.

A preliminary study to guide future research focus and assist in immediate leishmaniasis prevention and control policy decision making is underway in Isiboro-Secure National Park, Chaparé, Bolivia. Our objectives were to collect data on the prevalence of leishmaniasis in that area and evidence for peridomestic *Leishmania* transmission.

A survey was carried out during April–July 2007 in 2 communities in Isiboro-Secure National Park, San Gabriel (16°40′31′′S and 65°37′38′′W) and San Julian (16°41′59′′S and 65°38′10′′W). These 2 communities were selected because of local knowledge of disease in the community, their moderate degree of urbanization (i.e., ≈50% of the communities’ houses are clustered around the main access road), and the accessibility of the sites to the field team. In this area, CL is transmitted from April through October.

Households in both communities were visited by a team of experienced medical staff who interviewed heads of household to collect demographic data (sex, age) and diagnose the clinical condition of all present household members (presence/absence of CL lesions or scars, number of lesions, date of lesion onset) by using a standardized, pretested questionnaire. The study protocol was approved by the Ethical Committee Review Board of the World Health Organization (WHO). All patients with active cases were treated with meglumine antimoniate according to the standard protocol (*2*).

We surveyed 133 and 52 households in San Gabriel and San Julian, which represented 86% and 80% of the total households of the respective communities; 21 and 13 households, respectively, were visited but did not participate because the owners refused or were not present. Of the 965 persons surveyed, 488 (50.6%) were male and 476 (49.3%) were female; 9 (0.9%) had active CL lesions and 62 (6.4%) had CL scars. One person had ML, and 3 had evidence of past ML; all ML patients were male. Of those with CL lesions, all had 1 lesion only. The mean lesion size was 2.3 cm (range 1.5–3 cm), and the mean lesion duration (to survey date) was 5.6 months (range 1–11 months). The clinical CL lesions were parasitologically confirmed by microscopy (n = 4) or PCR (n = 8). Parasite culture was performed on patient isolates (n = 6), and *L.* (*V.*) *braziliensis* was identified and characterized as the etiologic agent of these CL cases.

Active lesion and scar prevalence were associated with male sex (lesions: Fisher exact test, odds ratio [OR] = 7.90 [95% confidence interval (CI) 1.01–169.09], p<0.05; scars: Yates-corrected χ^2^ test, OR = 3.05 [95% CI 1.65–5.71], p<0.001). Children <15 years of age were at lower risk of contracting the disease than those >15 years (lesions: Fisher exact test, OR = 0.19 [95% CI 0.01–1.46], p = 0.094; scars: Yates-corrected χ^2^ test, OR = 0.09 [95% CI 0.03–0.27], p<0.001) (Figure). Active lesion and scar prevalence were also associated with prolonged migration into the forest before the survey (lesions: Fisher exact test, OR = 28.10 [95% CI 3.49–184.29], p<0.01; scars: Fisher exact test, OR = 35.76 [95% CI 13.49–93.53], p<0.001).

**Figure:**
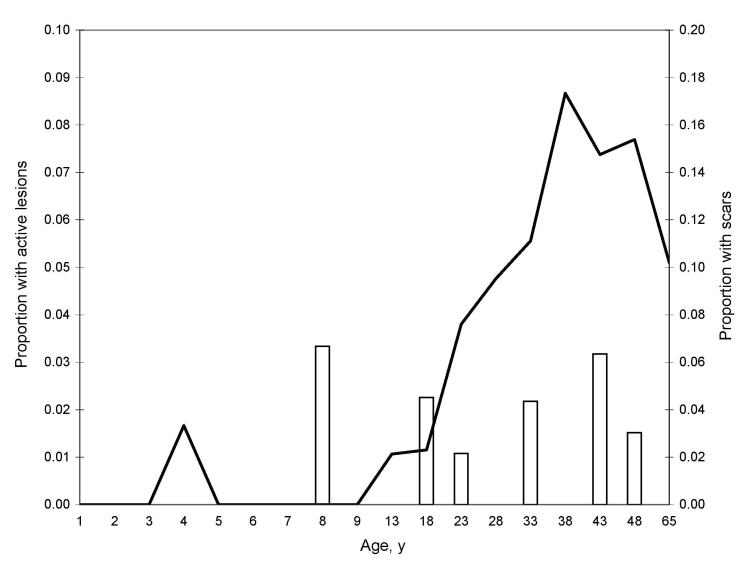
Age prevalence curve of persons with lesions (white bars) and scars (black line) from cutaneous leishmaniasis, Bolivia, 2007.

Whether the surveyed population is representative of the total population living in the study area is debatable. However, on the basis of current population figures (i.e., 16,000) and observed prevalence of CL, we estimate up to 1,440 CL cases in Isiboro-Secure currently. The low prevalence of active disease and scars indicates that *L.* (*V.*) *braziliensis* was introduced into Isiboro-Secure fairly recently, which is corroborated by the short median time since the cure of persons with CL scars (i.e., 7.5 years, range 0.4–30.5 years). Combined with the association of CL with male sex, age, and migration to the forest, we conclude that in Isiboro-Secure, most *L.* (*V.*) *braziliensis* transmission is sylvatic rather than peridomestic. This transmission pattern implies that prevention and control approaches that focus on the person (e.g., use of repellents, early treatment seeking) will most likely be more effective than approaches that focus on the household (e.g., indoor residual spraying with insecticides, insecticide-treated bednets).

Current analyses are underway to establish CL risk factors. Additionally, a prevention and control strategy adapted to the local context is being planned to minimize the population’s exposure to sandflies, prepare health professionals for adequate (per protocol) management of cases, and minimize the likelihood that *L.* (*V.*) *braziliensis* transmission becomes peridomestic.
